# The influence of a sustained 10‐day hypoxic bed rest on cartilage biomarkers and subchondral bone in females: The FemHab study

**DOI:** 10.14814/phy2.14413

**Published:** 2020-04-24

**Authors:** Adam C. McDonnell, Ola Eiken, Igor B. Mekjavic, Nik Žlak, Matej Drobnič

**Affiliations:** ^1^ Department of Automation, Biocybernetics and Robotics Jozef Stefan Institute Ljubljana Slovenia; ^2^ Department of Environmental Physiology Swedish Aerospace Physiology Centre Royal Institute of Technology Solna Sweden; ^3^ Department of Biomedical Physiology and Kinesiology Simon Fraser University Burnaby British Columbia Canada; ^4^ Department of Orthopaedic Surgery University Medical Centre Ljubljana Ljubljana Slovenia

**Keywords:** bed rest, bone density, cartilage, hypoxia, inactivity, serum markers, subchondral bone

## Abstract

This study assessed the influence of a 10‐day hypoxic bed rest on cartilage biomarkers and subchondral bone density across the patellofemoral joint (PFJ). Within clinical settings hypoxic tissue may arise in several types of disorders. Furthermore, a hypoxic environment is being considered for space flight habitats in the near future. Female participants (*N* = 12) participated in this study comprising three 10‐day interventions: hypoxic ambulation (HAMB), normoxic bed rest (NBR), and hypoxic bed rest (HBR). Venous samples were collected prior to (day −2: Pre) and during the intervention (days 2 and 5), immediately before reambulation (D11) and 24 hr post intervention (R1). Blood samples were analyzed for: aggrecan, hyaluronan, Type IIA procollagen amino terminal propeptide (PIIANP), and cartilage oligomeric matrix protein (COMP). Total bone mineral density (BMD) in eight regions (2 mm × 10 mm) across the PFJ was determined. The three interventions (HAMB, HBR, and NBR) did not induce any significant changes in the cartilage biomarkers of hyaluronan or PIIANP. Aggrecan increased during the HAMB trial to 2.02 fold the Pre value. COMP decreased significantly in both NBR & HBR compared to HAMB on D5. There were significant differences in BMD measured across the PFJ from cortical patellar bone (735 to 800 mg/cm^3^) to femur trabecular (195 to 226 mg/cm^3^). However, there were no significant changes in BMD from Pre to Post bed rest. These results indicate that there were no significant detectable effects of inactivity/unloading on subchondral bone density. The biomarker of cartilage, COMP, decreased on D5, whereas the addition of hypoxia to bed rest had no effect, it appears that hypoxia in combination with ambulation counteracted this decrease.

## Graphical abstract

Xxxx.

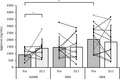

## 1 INTRODUCTION

The musculoskeletal system is a highly specialized tissue with complicated interrelationships. Of particular note is the synovial joint where articular cartilage is integrated with the subchondral bone and other connective tissues to maintain joint stability. The articular cartilage and subchondral bone provide an interrelated functional unit that is responsible for the gliding that takes place between the two surfaces with minimal friction and additionally dampens the forces that occur during locomotion (Hoemann, Lafantaisie‐Favreau, Lascau‐Coman, Chen, & Guzman‐Morales, 2012). These are tissues with a low metabolic activity; therefore, the impact of any clinical consequences may be delayed for months, or even years (Buckwalter & Mankin, 1998). However, preclinical detection of cartilage and subchondral bone turnover is possible with magnetic resonance (MR) and computer tomography (CT) imaging and/or by analyses of serum and urine biomarkers (Lotz et al., 2013; Roemer & Guermazi, 2012). Potential biomarkers of cartilage may indicate changes in cartilage metabolism within days of unloading (Liphardt et al., 2018) and earlier than with imaging techniques. Additionally, the same is apparent for bone whereby markers of bone resorption may indicate significant changes within days of inactivity (Rittweger et al., 2016).

According to the Carter and Wong chondral calcification theory (Carter & Wong, 2003), the response of chondrocytes to shear stress is calcification of adjacent cartilage, whereas compressive loading, resulting in a hydrostatic stress preserves the cartilage and prevents calcification. Cartilage thickness varies between joints and is a function of the forces developed within the joint. Thus, the regions subjected to the highest forces, such as the knee and hip, also have the thickest cartilage (Carter & Wong, 2003; Kurrat & Oberländer, 1978). Conversely, inactivity leads to thinning or degradation of the cartilage (Vanwanseele, Eckstein, Knecht, Stussi, & Spaepen, 2002; Vanwanseele, Lucchinetti, & Stussi, 2002), leading to the exudation from the joint of cartilage fragments. It has been demonstrated that the cyclic variation of hydrostatic pressure, within the physiological range of 2.8 to 10 MPa, that is applied to cartilage leads to the expression of cartilage‐specific genes and prevents thinning of the cartilage (for review see Carter and Wong (2003)).

Immobilization, limited weight‐bearing and bed rest are key triggers of articular cartilage atrophy (Eckstein, Hudelmaier, & Putz, 2006; Hinterwimmer et al., 2004; Hudelmaier, Glaser, Hausschild, Burgkart, & Eckstein, 2006; Liphardt et al., 2009; Vanwanseele, Eckstein, et al., 2002). Evans, Eggers, Butler, and Blumel (1960) reported that prolonged immobilization of rat knee joints caused major cartilage alterations, including matrix fibrillation, cleft formation, and ulceration. Bed rest is a ground‐based experimental model used to mimic the inactivity and unloading of the postural muscles and weight‐bearing bones that occurs during exposure to microgravity space flight, or reduced gravity during planetary exploration and therefore used to study the resultant adaptation of the physiological systems (Pavy‐Le Traon, Heer, Narici, Rittweger, & Vernikos, 2007). Ample literature demonstrates that strict continuous bed rest significantly alters cardiovascular and renal functions, as well as humoral status (Pavy‐Le Traon et al., 2007). Furthermore, it is well established that muscular atrophy and osteopenia have been identified as predominant musculoskeletal complications of human space habitation (Berg, Eiken, Miklavcic, & Mekjavic, 2007). In contrast, evidence demonstrating the responses of cartilage and subchondral bone under bed rest conditions is lacking (Liphardt et al., 2009, 2018).

Currently, the conditions on the International Space Station are normobaric and normoxic, therefore, experimental bed rest studies have been conducted in normoxic conditions (Pavy‐Le Traon et al., 2007). For logistical and safety reasons, future space habitats will maintain a hypobaric hypoxic atmosphere, to reduce the risk of decompression sickness during the preparation for extravehicular (EVAs) and extrahabitat activities (EHAs)(Bodkin, Escalera, & Bocam, 2006; Norcross et al., 2013). However, the pressure and gas boundaries have not been set, the current recommendations predict an equivalent altitude of approximately 1,500 m (8.2 psi/34% O_2_) to 2,500 m (7.6 psi/32% O_2_) (Norcross et al., 2005, 2013). For these reasons, we chose to exaggerate the level of hypoxia beyond these suggested levels in order to provide a significant physiological perturbation and investigate if there was an interaction effect between inactivity and hypoxia.

Whereas most research to date has focused on the process of musculoskeletal atrophy in males exposed to inactivity/unloading in normoxic conditions, the manner in which the hypoxic environments of future space habitats might affect the process of musculoskeletal atrophy in general, and in women in particular, remains to be resolved. A research program was established to address the effect of hypoxia on the processes of adaptation of various physiological systems to inactivity/unloading in male and female participants. Within the context of this program of research, the aim of this study was to assess the separate and combined effects of bed‐rest‐induced inactivity/unloading and hypoxia on subchondral bone in the patellofemoral joint (PFJ) and markers of cartilage metabolism in female participants.

## 2 METHODS

### 2.1 Ethical approval

This study was designed as a prospective, randomized, and controlled trial. The study protocol involved human participants and was approved by the National Medical Ethics Committee at the Ministry of Health (Republic of Slovenia), approval number: 205/2/11 and 88/04/12. The experimental procedures were conducted according to ESAs recommendations and conformed to the principles of the Declaration of Helsinki, except for registration in a database. Female participants were recruited and invited to attend individual interviews which were conducted by a panel of investigators. Exclusion criteria included: smoking, a medical record of respiratory, hematological, and cardiovascular problems, recent altitude exposure (<2 months), a history of mental illness and/or depression and the use of drugs or medications. The participants had no history of noteworthy musculoskeletal injuries or disease. Participants were given an extensive briefing regarding the study protocol and the possible risks involved were comprehensively explained prior to inclusion in the study. All participants were healthy recreationally active low‐land residents (<500 m) and gave their written informed consent for their participation in the study.

### 2.2 Bed rest protocol

This study was conducted in the hypoxic facility at the Olympic Sport Centre Planica (Rateče, Slovenia) and employed the experimental bed rest model. The participants were requested to take part in three 10‐d confinement interventions conducted at the Olympic Sport Centre Planica, situated at an altitude of 940 m. The interventions were separated by a 2‐month period. A total of 12 participants entered into the study and completed three interventions: (a) normobaric hypoxic ambulatory confinement (HAMB; P_I_O_2_ = 90 mmHg; *N* = 8), (b) normobaric normoxic bed rest (NBR; P_I_O_2_ = 133 mmHg; *N* = 11), and (c) normobaric hypoxic bed rest (HBR; P_I_O_2_ = 90 mmHg; *N* = 12). The hypoxic load (P_I_O_2_ = 90 mmHg) maintained during the HAMB and HBR interventions was equivalent to approximately 4,000 m. The participants were confined to one floor of the facility, where the ambient was maintained thermoneutral during all interventions.

In the horizontal bed rest trials (NBR and HBR), the participants maintained a horizontal position during all activities (i.e., reading, hygiene, feeding, lavatory, watching television, etc.). The participants were allowed to use one pillow for head support. Other than changing their body position from supine, prone, and lateral, no static or dynamic muscular contractions were permitted during the bed rest phase. Compliance with the bed rest protocol and participant safety was ensured via 24/7 closed circuit television monitoring and supervision by the medical staff. Each intervention comprised 5 days of baseline criterion data (basic core data, BCD) collection followed by the 10‐d intervention (HBR, NBR, or HAMB), which commenced at 09:00 on Day 1 and was terminated at 09:00 on Day 11, so that the cumulative exposure for each individual was exactly 240 hr. Postintervention BCD was collected during a 4‐d recovery period at the facility, please see Table 1.

**TABLE 1:**
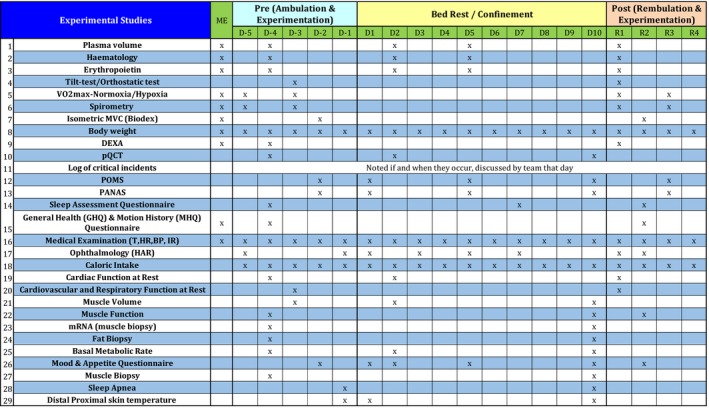
FemHab experimental overview. This figure presents the major experiments that took place during the female habitat research programme

The normobaric hypoxic environment within the Planica facility was established and maintained with a Vacuum Pressure Swing Adsorption (VPSA) system (b‐Cat, Tiel, The Netherlands) as described previously (McDonnell, Eiken, Mekjavic, & Mekjavic, 2014). Briefly, the VPSA system delivered O_2_‐depleted air to the designated rooms. The air in the rooms was sampled at 15‐min intervals and analyzed for O_2_ and carbon dioxide (CO_2_) content. As a safety precaution, the participants wore portable ambient O_2_ gas analyzers with audible alarms (Rae PGM‐1100) throughout the hypoxic interventions. During the HAMB intervention, the participants were encouraged to engage in their habitual routines and were allowed to move freely in the common hypoxic area. They also performed low‐intensity exercise sessions to mimic their prior habitual physical activity levels. Two 20‐min sessions were performed daily, one in the morning and one in the afternoon. To avoid monotony, the exercise modality (stepping, cycling, or dancing) was rotated daily. An individually tailored and standardized diet was provided to the participants throughout the interventions. The participants received the same food items on the same days of the respective interventions. Individualized energy requirements were calculated using the modified Harris–Benedict resting metabolic rate equation (Hasson, Howe, Jones, & Freedson, 2011). The participants were also administered vitamin D3 (1,000 IU daily) supplementation throughout the interventions and were encouraged to maintain their daily fluid intake above 28.5 ml/kg.

### 2.3 Hematology

Oestradiol and progesterone were assessed prior to (Pre), on the 2nd and 5th days of the intervention and on the 1st day of recovery (Post). The participants were fasted prior to the blood sampling. A blood sample was taken from the antecubital vein. Oestradiol and progesterone were measured with electrochemiluminescence immunoassay (ECLIA). The hormone concentrations were determined from both the calibration curve and the master curve reagent from the Roche Cobas e601 analyzer. The intraassay and interassay coefficient of variances for estradiol and progesterone are 6.1%, 2.9% and 7.0%, 4.9%, respectively. These data are presented in Table 2.

**TABLE 2:** Levels of oestradiol and progesterone measured 2 days before the interventions (Pre), Day 2, Day 5, and on completion (Post) of each intervention normobaric hypoxic bed rest (HBR: *n* = 12), normoxic bed rest (NBR: *n* = 11), and normobaric hypoxic confinement (HAMB: *n* = 8) campaigns

	HAMB				HBR				NBR			
	Pre	Day 2	Day 5	Post	Pre	Day 2	Day 5	Post	Pre	Day 2	Day 5	Post
Oestradiol (pg/ml)												
S1	18.31	31.06	63.61	453.80	8.72	49.88	117.90	81.50	266.30	115.50	133.50	83.65
S2	34.30	46.92	73.01	225.30	19.39	51.44	121.40	55.61	105.70	83.89	46.71	37.77
S3	8.24	5.00	10.30	5.00	5.00	5.00	5.00	5.11	5.00	34.92	5.60	5.00
S4	56.27	40.44	21.31	327.10	65.21	5.00	13.17	5.00	42.40	89.65	59.39	37.51
S5					15.18	5.01	5.01	40.31				
S6					63.14	111.60	111.60	32.79				
S7	11.98	31.95	84.06	160.20	152.60	32.96	19.49	19.80				
S8					5.00	5.00	5.00	5.00				
S9	166.40	81.00	111.50	12.30	7.13	5.00	6.81	5.00	6.51	7.72	5.13	5.00
S10	5.00	5.00	37.29	5.00	5.01	5.00	5.00	5.00	5.00	5.00	5.00	5.00
S11					5.00	28.16	49.74	6.73	5.00	37.87	11.45	5.00
S12	16.66	59.88	188.50	78.06	63.69	155.90	143.80	23.91	18.74	93.91	64.77	18.74
S14									5.00	5.00	5.00	5.00
S15									343.20	543.70	240.20	179.40
S16									23.74	49.43	92.08	69.56
Avg	39.65*	37.66*	73.70	158.35	34.59	38.33	50.33	23.81^#^	75.14	96.96	60.80	41.06^#^
*SD*	53.86	25.84	57.21	166.93	44.78	48.89	56.05	24.80	118.58	153.18	73.13	53.65
Progesterone (ng/ml)												
S1	0.90	0.39	0.49	0.51	0.34	0.31	0.35	1.63	0.26	0.63	5.86	3.63
S2	0.90	0.82	0.76	0.80	0.56	0.75	0.56	3.37	7.44	4.70	1.49	0.60
S3	0.46	0.49	0.38	0.42	0.32	0.30	0.52	0.42	0.22	0.14	0.37	0.33
S4	0.74	1.02	0.69	1.03	1.60	0.75	0.64	0.51	9.37	1.75	0.76	0.94
S5					0.99	1.04	1.04	0.63				
S6					3.54	14.44	14.40	1.60				
S7	0.63	0.58	0.57	0.41	16.80	2.49	0.73	0.56				
S8					0.36	0.29	0.23	0.28				
S9	0.91	1.62	7.43	1.14	1.02	1.00	0.83	0.24	1.02	1.06	0.85	0.93
S10	0.25	0.24	0.47	0.29	0.25	0.22	0.25	0.95	0.30	0.20	0.28	0.20
S11					0.73	0.54	0.43	0.72	0.63	0.58	0.63	0.51
S12	0.40	0.50	0.59	3.54	2.24	7.57	5.99	0.61	0.70	0.76	0.71	0.73
S14									0.86	0.95	0.96	0.86
S15									0.84	1.75	8.55	6.10
S16									1.43	1.53	1.46	2.92
Avg	0.65	0.71	1.42	1.02	2.40	2.47	2.16	0.96	2.10	1.28	2.12	1.85
*SD*	0.26	0.44	2.43	1.07	4.64	4.29	4.16	0.88	3.17	1.27	2.78	1.97

Note

Participants 3, 5, 9, 10, 11, and 14 (highlighted in red) were using hormonal contraception, whereas the remaining participants were naturally cycling.

Significant differences (*p* < .05): * versus R1, # versus HAMB

### 2.4 Cartilage serum markers

Venous blood samples (10 ml) were obtained in each of the interventions at the following five time points: two days before the intervention (Pre), on the 2nd and 5th day of the intervention (D2, D5) and Day 11 (just prior to reambulation (D11)) and 24 hr following the intervention (R1). All blood samples were standardized for time and day, thus they were drawn from an antecubital vein in the morning (07:00) following an overnight fast; at all time points (Pre, D2, D5, D11, and R1), the participants remained in bed and supine prior to the venepuncture and until the completion of the blood sampling. A 4 ml sample was immediately transferred to a nearby clinical laboratory and the remaining venous blood was centrifuged (10 min at 3,500 rpm; 4°C), stored in six 400 μL aliquots and immediately frozen at −80°C for subsequent analyses performed ≤12 months after the completion of the intervention. All samples were analyzed in duplicate. Four serum cartilage biomarkers were analyzed according to the manufacturer's manuals:

- Aggrecan is the predominant and largest proteoglycan species in articular cartilage. It is composed of a core‐protein of 210 kDa, to which sulfated polysaccharide chains of chondroitin sulfate and keratan sulfate are linked. A solid phase enzyme‐amplified sensitivity immunoassay (PG‐EASIA, DIAsource ImmunoAssays, Belgium) was used for the detection of aggrecan in the blood samples. This antibody detects recently synthesized and intact molecules of aggrecan and it is used to detect its turnover (El‐Arman, El‐Fayoumi, El‐Shal, El‐Boghdady, & El‐Ghaweet, 2010).
- Hyaluronan is one of the main components of the cartilage matrix as well as the synovial fluid. An immunoassay TECO® Hyaluronic Acid PLUS ELISA (TECO medical group, Switzerland) was used for the analysis according to the manufacturer's recommendation. Hyaluronan levels correlate with the degree of synovial proliferation and the length of osteophytes, but not with the femoral cartilage thickness and it is therefore considered as a marker of inflammation (Elliott et al., 2005).
- Type IIA procollagen amino terminal propeptide (PIIANP) is cleaved off and released into body fluids during collagen formation. It is therefore considered as a marker for collagen synthesis (Martel‐Pelletier et al., 2017). PIIANP assay detects the cysteine‐rich globular sequence in PIIANP. Cartilage damage following trauma or disease onset rapidly leads to the increased synthesis of matrix components to compensate for losses. Type II collagen is synthesized by the chondrocytes as type II procollagen. During collagen formation, the amino and carboxy‐terminus propeptides are cleaved off and released into body fluids. Human PIIANP ELISA kit (EMD Millipore Corporation, USA) was used in the study.
- Cartilage oligomeric matrix protein (COMP) is a noncollagenous extracellular matrix protein synthesized by cartilage, but also by other cell types including synovial cells and osteoblasts. It is released during cartilage degradation, but high serum COMP might also indicate synovial inflammation (Andersson et al., 2006). Human Cartilage Oligomeric Matrix Protein ELISA (BioVendor—Laboratorní medicína a.s., Czech Republic) was used according to the manufacturer's recommendations to identify changes in COMP.

### 2.5 Bone density pQCT analysis

**FIGURE 1:**
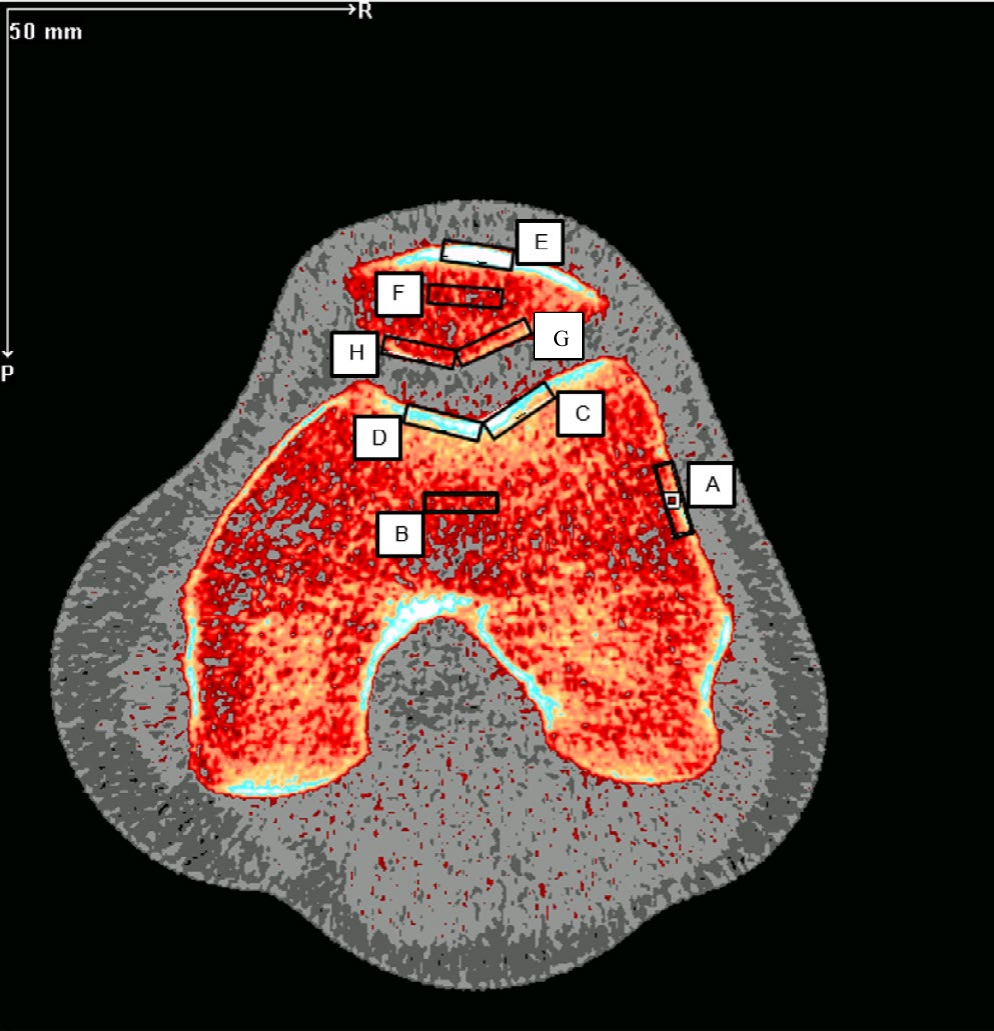
Subchondral bone density regions of interest, as derived from the pQCT images of the patellofemoral joint. Eight regions of interest (length 2 mm × width 10 mm) were defined: A: femoral cortical (cortical bone on medial condyle cortex), B: femoral trabecular (central part of condyles), C: trochlea lateral facet (subchondral bone just below the lateral trochlea groove), D: trochlea medial facet (subchondral bone just below the medial trochlear groove), E: patella cortical (cortical bone on ventral patella surface), F: patella trabecular (central part of patella), G: Patella lateral facet (subchondral bone just below the lateral patella facet) and H: patella medial facet (subchondral bone just below the medial patella facet)

The morphology of subchondral bone was analyzed by peripheral quantitative computer tomography (pQCT, XCT3000 Stratec Medizintechnik), which was conducted onsite at the bed rest facility. Participants were positioned in the supine position with their left leg (nondominant) fully extended and positioned within the device. The foot was fixed to a custom plastic holder and secured with an elastic strap, whereas the thigh was fixed with a supporting holder provided by the manufacturer, thus improving the repeatability of the scanning location. Initial scanning was performed 3 days before the intervention (Pre) and the same scanning protocol was repeated on the 1st day following the 10 day intervention (Post). On a transversal CT slice across the distal femoral epiphysis (4% of femoral length from the knee joint line), the mid portion of the patellofemoral joint (PFT) was visible. Eight regions of interest (ROIs, length 2 mm × width 10 mm) were defined, as shown in Figure 1:

- Femoral cortical (cortical bone on medial condyle cortex),
- Femoral trabecular (central part of condyles),
- Trochlea lateral facet (subchondral bone just below the lateral trochlea groove),
- Trochlea medial facet (subchondral bone just below the medial trochlear groove),
- Patella cortical (cortical bone on ventral patella surface),
- Patella trabecular (central part of patella),
- Patella lateral facet (subchondral bone just below the lateral patella facet), and
- Patella medial facet (subchondral bone just below the medial patella facet).

A blinded researcher individually placed each ROI on the scan and applied the manufactures’ bone mask. Thereafter, the results were exported to Excel for analysis. Bone mineral density (BMD) in each ROI identified above was analyzed by an integrated pQCT software (Stratec Medizintechnik software version 6.20B, Pforzheim, Germany). The precision error of the XCT 3000 has been reported to be as low as 0.32% (Swinford & Warden, 2010), 0.4% (Groll, Lochmuller, Bachmeier, Willnecker, & Eckstein, 1999; Weatherholt et al., 2015), and 0.5% (Rittweger & Felsenberg, 2009).

### 2.6 Statistical analysis

Data regarding the serum cartilage markers and regional BMD are presented as means and standard deviations. The Shapiro–Wilk test was carried out to assess for normal distribution. Repeated measures general linear model (GLM) was used to assess significance between measurements of each intervention and different time points for cartilage serum markers. Bonferroni's multiple comparisons test was used to compare values in individual time unit pairs when differences were established in the first step. One‐way ANOVA followed by a Tukey post hoc test was used to compare Pre to Post regional BMD between the interventions (NBR, HBR, HAMB) at the different locations  of the PFJ. SPSS statistics Version 23 was used for the analysis. *p* values ≤0.05 were considered statistically significant.

## 3 RESULTS

The participants physical characteristics were as follows: stature = 169 ± 6 cm; body mass = 59.5 ± 8.8 kg; Body Mass Index (BMI) = 20.9 ± 2.5 kg/m^2^; body fat = 28.8 ± 4.5%, maximal oxygen consumption (VO_2max_) = 41 ± 3.8 ml kg^−1^ min^−1^.

### 3.1 Hormone response

Data presented in Table 2 indicate that several of the participants menstruated during the data collection period or within the intervention. Six of the participants (3, 5, 9, 10, 11, and 14) were taking hormonal contraceptives, whereas the remainder were naturally cycling. The following participants experienced menses during the interventions: HAMB participants 1, 2, 7, 9, 10, and 12; HBR: 1, 2, 5, 6, 7, 11, and 12; and NBR: 3, 9, 11, 12, 15, and 16. These participants are marked with a dashed line in Figure 3. Table 2 indicates significant differences noted in oestradiol in Pre and Day 2 of the HAMB intervention compared to Post. Additionally, Post HAMB was different to both HBR and NBR for the same time point. Progesterone tended to be lower at the Pre time point in HAMB compared to both HBR (*p* = .1) and NBR (*p* = .08) although this difference did not reach statistical significance.

### 3.2 Cartilage serum markers

**FIGURE 2:**
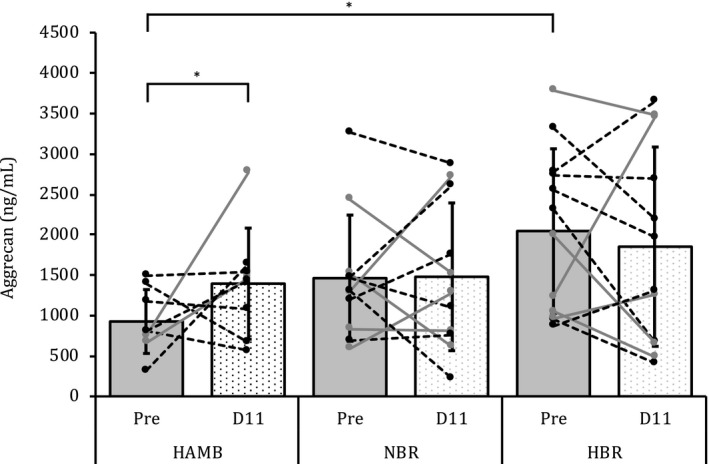
Serum cartilage markers (ng/ml) aggrecan, hyaluronan, PIIANP, and COMP from the female participants prior (Pre) to, during (Day 2 and 5), and after (D11 and R1) the three interventions: normoxic bed rest (NBR), hypoxic bed rest (HBR), and hypoxic ambulation (HAMB). Results are presented as estimated marginal means. Markers of significance: * Denotes a difference between HBR and HAMB on the same intervention day. ^§^Denotes a difference within condition

Baseline values for the biomarkers of cartilage metabolism analyzed for each condition were compared and were found not to be different. The exception was aggrecan. There was a significant difference in the serum marker of aggrecan at Pre in the HAMB condition compared to HBR, *p* < .05. No other baseline differences were noted. There was a progressive increase in aggrecan from the baseline value so that D11 was significantly different compared to Pre in the HAMB condition and thereafter decreased toward baseline values on R1, *p* < .05, see Figure 2. There were no other changes noted in aggrecan.

**FIGURE 3:**
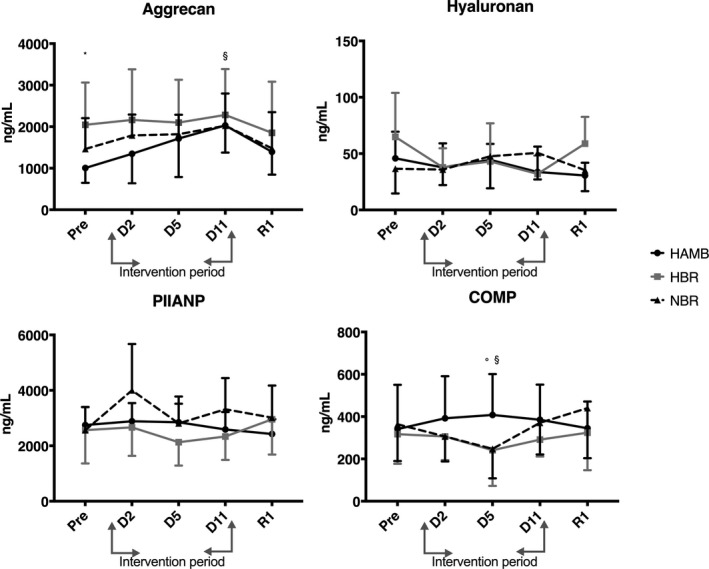
Individual responses of aggrecan prior to (Pre) and on D11 of the intervention. The dashed lines are the participants who menstruated at one of the time points in the figure. *denotes a difference between HAMB Pre to D11 and HAMB Pre to HBR Pre

As there were differences noted in the baseline values (between condition) of aggrecan and compared to D11, this information is presented in an additional format to provide clarity to the reader, Figure 3. The individual responses are depicted over the group average.

The biomarker COMP significantly decreased from Pre to D5 in NBR (*p* = .047), but not in HBR (*p* = .12). This led to a significant difference between the HAMB and both bed rest (NBR & HBR) conditions on D5, *p* = .002.

There were no significant effects of any of the interventions on the serum levels of the biomarkers Hyaluronan and PIIANP.

### 3.3 Bone density pQCT analysis

Bone density, as would be expected, differed significantly across the PFJ between the eight analyzed ROIs. Cortical patellar bone exhibited the greatest density (735 to 800 mg/cm^3^), followed by both trochlear facets (495 to 548 mg/cm^3^), both patellar facets (293 to 433 mg/cm^3^), femoral cortical bone (273 to 288 mg/cm^3^), trabecular bone of the patella (213 to 233 mg/cm^3^), and lastly the trabecular bone of the femur (195 to 226 mg/cm^3^). There were no statistical differences noted in the ROIs for bone density as a result of the interventions. Details are provided in Table 3.

**TABLE 3:** Total bone mineral density (mg/cm^3^) across PFJ measured by pQCT in female participants during the three interventions (HAMB, NBR, HBR)

Joint region	Timing	HAMB (*N* = 8); mg/cm^3^	NBR (*N* = 11); mg/cm^3^	HBR (*N* = 12); mg/cm^3^
A: femoral cortical	Pre	287.7 (51.8)	276.2 (49.3)	273.1 (46.2)
	Post	284.3 (52.8)	285.6 (54.6)	277.0 (42.4)
B: femoral trabecular	Pre	204.9 (66.7)	195.4 (49.0)	216.5 (61.0)
	Post	202.9 (57.9)	197.5 (56.5)	226.3 (74.7)
C: trochlea lateral facet	Pre	544.7 (109.4)	510.1 (102.0)	541.3 (82.5)
	Post	529.6 (102.9)	517.9 (90.3)	547.8 (91.8)
D: trochlea medial facet	Pre	512.0 (119.6)	495.8 (111.3)	513.3 (102.2)
	Post	515.9 (101.4)	519.3 (103.4)	530.2 (100.2)
E: patella cortical	Pre	792.8 (99.5)	767.4 (128.3)	799.7 (88.0)
	Post	771.8 (112.3)	734.9 (94.0)	783.8 (97.9)
F: patella trabecular	Pre	219.2 (49.0)	232.9 (40.0)	224.6 (36.6)
	Post	212.7 (36.8)	232.3 (38.0)	229.9 (35.9)
G: Patella lateral facet	Pre	382.3 (175.4)	327.2 (122.1)	374.5 (100.7)
	Post	362.9 (161.5)	297.3 (117.1)	311.4 (111.6)
H: patella medial facet	Pre	433.4 (251.4)	339.0 (179.6)	355.5 (127.0)
	Post	386.6 (215.2)	299.5 (170.1)	292.5 (133.1)

Note

Abbreviations: HAMB, hypoxic ambulatory; HBR, hypoxic bed rest; NBR, normoxic bed rest.

## 4 DISCUSSION

The principle findings from this study with female participants are that the serum markers were affected differently by each condition (HAMB, HBR, and NBR), albeit with no significant changes in the serum markers hyaluronan and PIIANP. COMP was significantly lower on day 5 of both bed rest settings compared to ambulation (HAMB). There was no change in subchondral bone density measured with pQCT as a result of any of the conditions.

### 4.1 Inactivity and unloading

There is substantial evidence in the literature that cartilage and subchondral bone are influenced by activity or lack thereof. This becomes clinically important during periods of immobilization, unloading, or prolonged bed rest (Eckstein et al., 2006; Hinterwimmer et al., 2004; Hudelmaier et al., 2006; Vanwanseele, Eckstein, et al., 2002). This type of bone and joint disuse atrophy may be counteracted by exercise that is aimed at strengthening the muscles, while maintaining movement within the joints range of motion and by providing early protective weight‐bearing below the threshold of local tissue damage (Buckwalter, 1995). There is a paucity of data regarding cartilage turnover during bed rest and in particular hypoxic bed rest and none available for females. While biomarkers of cartilage react quickly, actual articular cartilage changes are slower and currently not the primary focus of space life sciences research. To our knowledge, there is limited evidence regarding the effect of bed rest on cartilage (Liphardt et al., 2009, 2018). Liphardt et al. (2018) measured serum levels of COMP on days 14 and 21 of bed rest and noted that COMP decreased by 16% after 24h of bed rest and a further 5% by day 14 of bed rest, with no further change by Day 21. Thereafter, the values returned to normal levels on the first day of recovery.

Immobilization and unloading of the weight‐bearing bones does not appear to have the same effect in animal models. Using the hindlimb suspension model, O'Connor (1997) noted that after 28‐days of joint unloading there was no change in the articular cartilage thickness in rats. However, there was a significant alteration in the constituents, specifically, an increase in the thickness of the calcified cartilage and a concomitant decrease in uncalcified cartilage thickness. In contrast to these findings, there were no differences in the thicknesses of the calcified and uncalcified layers when the unweighted limb was also immobilized (Muhlbauer, Lukasz, Faber, Stammberger, & Eckstein, 2000). O'Connor (1997), concludes that unweighting a mobile joint increases calcification, whereas restriction of motion initiates resorption of the chondro‐osseous interface. In humans, in the absence of normal joint loading and movement, there is significant thinning of cartilage (Vanwanseele, Eckstein, et al., 2002). The largest changes in cartilage thickness can be seen at the medial aspect of the tibia, observed 6 months after a spinal cord injury, but not in the lateral tibia (Vanwanseele, Eckstein, et al., 2002). The separate and combined contributions of weight‐bearing and joint movement, on the thickness of articular cartilage in humans remains as yet unresolved. Furthermore, neither aggrecan nor COMP serum levels appear to be highly correlated with cartilage thickness (Bricca, Juhl, Grodzinsky, & Roos, 2017; Erhart‐Hledik et al., 2012), suggesting future studies include both biomarker analysis and high‐quality imaging techniques to provide an entire picture of cartilage status. With regard to astronauts, they are physically active and therefore maintain joint movements, whereas the limbs are unloaded during spaceflight and therefore may be unlikely to stimulate the same cartilage resorption seen in spinal cord injury or animal models.

Subchondral bone provides an interface with the overlying articular cartilage, defects noted in the cartilage may also be detected in the bone. Biomechanical stress is a key requirement to stimulus subchondral bone and articular cartilage in order to maintain joint health and the removal of such a stimulus is crucial in the reduction of joint integrity (Fitzgerald, Endicott, Hansen, & Janowitz, 2019; Hlaing & Compston, 2014; Hoemann et al., 2012; Sun, 2010). With the removal of this stress, that is bed rest, there is a rapid loss of proteoglycans from cartilage and a thinning of the cortical bone (Rittweger et al., 2009). The time course of this bone loss during bed rest experiments is approximately 0.5% per week of inactivity (Berg et al., 2007; Iwamoto, Takeda, & Sato, 2005; Ohshima, 2010). We have previously reported in male participants no change in bone mineral content (BMC) of the tibia or femur (McDonnell, Eiken, Frings‐Meuthen, Rittweger, & Mekjavic, 2019). The short timeline of the physiological withdrawal of mechanical loading is likely insufficient for us to observe with an imaging technique, any structural changes in the bone density or content following the functional alterations. Although, Armbrecht et al. (2011) indicate changes following 15 days in females. One potentially confounding factor is that while the participants are under strict bed rest, the patellofemoral joint may still experience loading due to the participants daily movement in bed, that is, turning from prone to supine, or lifting hips to use the bed pan. In line with Wolfs law that mechanical force is the key to bone formation, it may be theorized that this small level of quadricep and hamstring activity may apply enough pressure on or throughout the joint and likely affect the subchondral bone, slowing deterioration.

While it is important to conduct 10‐day bed rest studies as is currently recommended by ESA, the addition of hypoxia to bed rest, however, is novel and we are not aware of the publication of pQCT data in females in a hypoxic environment. There is some evidence in vitro with the use of animal cells, albeit in extreme hypoxia of 1 to 2%, that the hypoxic ambient stimulates osteoclast formation (Arnett, 2010; Genetos et al., 2010). Similar action has been indicated by Utting, Flanagan, Brandao‐Burch, Orriss, and Arnett (2010) with the use of cultured human cells. However, this level of hypoxia is improbable *in vivo* in humans. A low oxygen tension will stimulate an increase in the differentiation of monocytes, macrophage leading to increased numbers of mature osteoclasts. *In vitro* studies have demonstrated that hypoxia may act to both inhibit and stimulate osteoblast activity (Knowles, 2015; Knowles & Athanasou, 2009; Utting et al., 2010). Thus, it is currently unknown, particularly in the short term, whether hypoxia will exacerbate osteoclast action and bone resorption or mitigate the degradation of bone in bed rest through increased osteoblast proliferation and differentiation. The mechanisms for such action through HIF regulation and RANKL require future investigation under bed rest and hypoxia, (for review see Knowles, 2015). Thus, although any significant changes may only be observed following 15 days of normoxic bed rest Armbrecht et al. (2011) and following 21 days of hypoxic bed rest (Rittweger et al., 2016), the use of pQCT during shorter duration bed rest studies, particularly when an additional intervention is introduced, namely hypoxia, is pertinent to the established time line of bone mineral loss. Nevertheless, the current results are consistent with a normoxic environment.

### 4.2 Cartilage synthesis and degradation

The responses of the aggrecan marker of cartilage turnover are not clear as the baseline values were different, however, such a change in HAMB would be suggestive of increased cartilage formation during the early period of the hypoxic ambulatory condition. Although such a response is not limited to the knee joint, since serum markers reflect whole body cartilage synthesis, including that of the vertebral column, all large joints, and rib‐chondral junctions. Concomitant with this potential increase in cartilage formation, the observed decline in COMP levels up to D5 of bed rest, suggests less cartilage degradation. A similar COMP profile was reported by Liphardt et al. (2018), however, they also observed an additional 5% decrease by day 14 of bed rest. Whether this difference might be attributed to the fact that our study included female participants as opposed to males, remains to be settled. Furthermore, large individual variation can play a role in making significant physiological effects. These individual responses may be viewed in Figure 3. There is no discernible pattern to the direction of change as a result of either intervention or through the effects of the hormonal cycle. There is evidence that progesterone plays a role in regulating aggrecan and aggrecanases and therefore it is crucial to monitor and coordinate the menstrual cycle for future work in this area (Wen, Zhu, & Leung, 2013; Yasuo, Yamaguchi, & Kitaya, 2010). Yasuo et al. (2010) report significant changes in the levels of serum aggrecan as a result of the menstrual cycle with peaks during the mid to late secretory phase compared to the proliferative phase. Please see Table 2 to refer to details of the participants’ hormone levels throughout this study. While progesterone was not significantly lower in HAMB compared to HBR and NBR, a low level of progesterone maybe associated with a reduced serum aggrecan (Yasuo et al., 2010), although this is not evident in our participants. The manufacturer (DIAsource) of the PG‐EASIA kit report in their instructions that a typical range of aggrecan for adults in serum is 1,000 to 4,400 ng/ml with an average of 2,800 ng/ml in adults. They did not specify a difference as a result of gender. Very low levels of aggrecan of 78 ng/ml have been reported in teenage participants with and without polycystic ovary syndrome (PCOS) (Tola, Koroglu, Yalcin, & Oral, 2018). Although no significant difference was found between groups, those with PCOS tended to exhibit higher levels of aggrecan secretion during the follicular phase. In contrast, within our data the lowest aggrecan recorded was 320 ng/ml and highest 4,296 ng/ml. Our group data fit within the typical expected range, although there are outliers.

Weitoft, Larsson, Saxne, and Rönnblom (2005) studied levels of COMP in rheumatic patients with knee synovitis after intra‐articular glucocorticoid treatment. Patients were randomized to 24‐hr bed rest or to normal activity following the treatment. After a glucocorticoid injection (Weitoft et al., 2005), COMP levels decreased in both groups, but significantly more in the bed ridden patients. This could suggest either a transient protective effect of bed rest on the articular cartilage, or a lower clearance of COMP from the knee joint due to the restricted activity. The significant difference noted in this study on D5 between the ambulatory group (HAMB) and bed rest groups (HBR and NBR) could suggest either a transient protective effect of bed rest on articular cartilage, or a lower clearance of COMP from the knee joint. The activity of the HAMB group may also be responsible for an increased clearance of COMP and aggrecan from the joint. The former may partially explain the decrease by day 14, but no further decrease by Day 21 of bed rest (Liphardt et al., 2018).

Experience from joint distraction arthroplasty suggests that this, somewhat obscure surgical technique for treatment of knee or ankle osteoarthritis, not only diminishes patients’ symptoms, but also induces cartilage repair (van der Woude et al., 2017). It is clear that physical activity has a significant physiological effect on cartilage thickness (Bricca et al., 2017), compared to spinal cord‐injured individuals healthy humans exhibit thicker cartilage at the trochlear notch, medial and lateral condyle, and femoral cartilage (Yilmaz, Demir, Ozyoruk, Kesikburun, & Guzelkucuk, 2016). Although, following a review of the literature, Bricca et al. (2017) were unable to establish a link between aggrecan and cartilage thickness, they did suggest that activity level is nonlinearly linked with cartilage composition rather than thickness, that is, joint health. Aggrecan is responsible for the absorption of water under normal loading providing a buffering surface to absorb impact within the joint. Under sedentary conditions, aggrecan may begin to degrade with cleaved particles entering the synovium. Given the current physical activity levels in the developed world, it may be more appropriate to state that physical inactivity has profound detrimental effects on cartilage thickness (Yilmaz et al., 2016). Animal studies have clearly demonstrated the negative effect of prolonged unloading and immobilization (Haapala et al., 1999). Nomura et al. (2017) noted that while the murine joint surface remains intact following unloading and immobilization, total and uncalcified cartilage thickness decrease. It is possible to measure decreased aggrecan levels and increased aggrecanase activity in uncalcified cartilage layers.

### 4.3 Hypoxia

The effect of hypoxia on cartilage has not yet been studied clinically. In this study, we potentially observed a significant increase in Aggrecan (reflecting cartilage formation) in ambulatory participants exposed to hypoxia. There is currently a growing interest in Hypoxia‐Inducible Factors (HIFs), which are transcriptional factors and key regulators of the cellular response to hypoxia (Fernandez‐Torres, Zamudio‐Cuevas, Martinez‐Nava, & Lopez‐Reyes, 2017). Particularly, HIF‐1α has been shown to have a protective effect in the maintenance of articular cartilage (Thoms, Dudek, Lafont, & Murphy, 2013). Furthermore, chondrocytes in human cartilage are exclusively adapted to hypoxia and use it to regulate tissue‐specific metabolism (Thoms et al., 2013). We believe that the clinical effects of intermittent hypoxia on articular cartilage warrant further investigation.

### 4.4 Space flight

Astronauts participating in long‐term space missions experience musculoskeletal atrophy. Niehoff et al. (2016) reported an increase in serum COMP levels of astronauts 7 and 30 days after return to Earth. In the absence of in‐flight values, they concluded that the postflight increase observed on returning to Earth is indicative of the re‐instated mechanical load. COMP would appear to be sensitive to exposure to microgravity and worthy of further study. As a consequence of the deleterious changes associated with space flight, ground‐based research studies have begun to focus on the efficacy of countermeasures. One such countermeasure of purported significance is vibration resistive exercise. Liphardt et al. (2009) reported that vibration training partially prevented the loss of cartilage thickness in the tibia, but did not have an effect on the serum COMP levels. Their observed reduction in serum COMP levels over the course of a 14‐day 6° head‐down tilt bed rest is similar to the observed trend of decreasing COMP in this study (Liphardt et al., 2009). The observation of a significant difference in cartilage thickness between participants receiving vibration training and those who did not, concomitant with no change in COMP levels, raises some concern regarding the sensitivity of COMP in reflecting the integrity of the collagen network or whether there is a time point more reflective of the activity of circulating COMP which has not been identified to date. Current and future space flight is and will be composed of mixed gender space farers, with this in mind, it is imperative to include females in space life science research. Within the scope of this study, while we did not control for the menstrual cycle in our participants, the possibility exists that, menstruation may affect the serum cartilage markers’ profiles, although there is no published evidence of this potential effect or lack thereof, it should be considered.

### 4.5 Limitations

The results of this study need to be interpreted in view of certain limitations. The 10‐day time frame of the bed rest may be too short to observe any changes in subchondral bone density. The pQCT device utilized during the experiment enabled only traversal image acquisition, therefore the PFJ of the nondominant leg was used for the analysis of subchondral bone density. Better insight into subchondral bone changes may be obtained, if the tibio‐femoral joint surfaces are also used. Although the PFJ is not directly loaded in full knee extension, it withstands high loads during locomotion. Individual variation, the menstrual cycle and plasma volume reduction may mask changes occurring in serum markers. Finally, previous results from a 21‐day hypoxic bed rest in men, indicate that plasma volume (calculated not measured) was reduce following bed rest (cf. (Keramidas et al., 2016). It seems likely that a reduction in plasma volume occurred within that 10‐day period in men. Similarly, the female participants in this study lost plasma volume in both bed rest conditions, HBR: −15% and NBR: −8% (Mekjavic et al., under review). Thus, the present data regarding the serum markers, may be somewhat underestimated for decrements, for example, COMP values and somewhat overestimated for increments, for example, aggrecan values.

## 5 CONCLUSIONS

In females, 10 days of bed rest while exposed to a hypoxic environment did not clearly indicate a trend of changes in the serum levels of markers for cartilage synthesis and degradation or inflammation across time and condition. This time period of bed rest or inactivity is likely too short to measure physiologically significant changes in bone density in the PFJ, whereas some biomarkers of cartilage metabolism may identify changes. The increase in aggrecan noted in the HAMB trial suggests an increased turnover of cartilage and possibly a protective effect of hypoxia during activity compared to inactivity, however, caution showed be applied here as the baseline values were different.

## CONFLICT OF INTEREST

The authors declare no conflicts of interest.

## AUTHOR CONTRIBUTIONS

The work was conducted at the Institute Jozef Stefan laboratory in Planica, Slovenia. 1. Conception or design of the work (MD, AMCD, IBM, OE). 2. Acquisition, analysis, or interpretation of data for the work (MD, AMCD, IMB, NZ, OE). 3. Drafting of the work or revising it critically for important intellectual content (MD, AMCD, IMB, NZ, OE). 4. Approved the final version of the manuscript (MD, AMCD, IMB, NZ, OE). 5. Agree to be accountable for all aspects of the work in ensuring that questions related to the accuracy or integrity of any part of the work are appropriately investigated and resolved. (MD, AMCD, IMB, NZ, OE). 6. All persons designated as authors qualify for authorship, and all those who qualify for authorship are listed. (MD, AMCD, IMB, NZ, OE).
